# Polymorph evolution during crystal growth studied by 3D electron diffraction

**DOI:** 10.1107/S2052252519016105

**Published:** 2020-01-01

**Authors:** Edward T. Broadhurst, Hongyi Xu, Max T. B. Clabbers, Molly Lightowler, Fabio Nudelman, Xiaodong Zou, Simon Parsons

**Affiliations:** a EaStCHEM School of Chemistry and Centre for Science at Extreme Conditions, The University of Edinburgh, King’s Buildings, West Mains Road, Edinburgh EH9 3FJ, UK; b Department of Materials and Environmental Chemistry, Stockholm University, Stockholm SE-106 91, Sweden

**Keywords:** crystallization, polymorphism, cryoTEM, electron diffraction, 3DED, glycine

## Abstract

3D electron diffraction (3DED) has been used to follow polymorph evolution in the crystallization of glycine from aqueous solution. The three polymorphs of glycine which exist under ambient conditions follow the stability order β < α < γ. The least stable β polymorph forms within the first 3 min, but this begins to yield the α-form after only 1 min more. Both structures could be determined from continuous rotation electron diffraction data collected in less than 20 s on crystals of thickness ∼100 nm. Even though the γ-form is thermodynamically the most stable polymorph, kinetics favour the α-form, which dominates after prolonged standing. In the same sample, some β and one crystallite of the γ polymorph were also observed.

## Abstract 2

3D electron diffraction has been used to follow polymorph evolution during the crystallization of glycine from pure water. All three known polymorphs formed sequentially in samples taken from the same solution and allowed to crystallize *in situ* at times between 3 and 60 min. Crystal structures were obtained for all three phases.

## 1. Introduction   

Polymorphism, the formation of different crystal structures by a single compound, is of critical importance in applications such as opto-electronics, energy storage and, most famously, pharmaceuticals. It is a common feature of organic solids, with a likely occurrence rate of at least 50%, rising to 74% for a set of materials for which extensive polymorph screening had been carried out by Roche (Cruz-Cabeza *et al.*, 2015 ▸). Its importance arises because different solid forms usually have different physical properties such as solubility, morphology or tabletting characteristics. Moreover, transitions between polymorphic forms can occur on storage. Infamous examples, such as Ritonavir (Bauer *et al.*, 2001 ▸; Bučar *et al.*, 2015 ▸), demonstrate that insufficient characterization of polymorphism can lead to life-threatening interruptions of drug therapies and huge commercial losses. Polymorph screening is thus a vital stage of development, but it is also an expensive and time-consuming activity.

Recent work on inorganic systems (Pichon *et al.*, 2008 ▸; Walker *et al.*, 2017 ▸) has demonstrated that the rapidly developing technique of cryo-transmission electron microscopy (cryoTEM) can be used to monitor the crystallization of calcium carbonate from solution, showing how initially formed amorphous calcium carbonate particles cluster together and then transform into aragonite or calcite. Amorphous calcium carbonate is metastable with respect to aragonite and calcite, and this observation also illustrates the tendency for thermodynamically higher-energy polymorphs to form in the early stages of crystallization (Ostwald’s Rule of Stages; Bernstein, 2010 ▸). Micrometre-sized crystals that are too small for X-ray diffraction are suitable for structure determination by 3D electron diffraction (3DED), also referred to as microcrystal electron diffraction, continuous rotation electron diffraction (cRED) or electron diffraction tomography (EDT) (Wan *et al.*, 2013 ▸; Palatinus *et al.*, 2015 ▸; Colmont *et al.*, 2016 ▸; Gruene *et al.*, 2018 ▸; Jones *et al.*, 2018 ▸; Andrusenko *et al.*, 2019 ▸; Brázda *et al.*, 2019 ▸; Gemmi *et al.*, 2019 ▸; Xu & Zou, 2019 ▸). The aim of this report is to illustrate how a combination of the methodologies used in cryoTEM and 3DED with *in situ* crystal growth can be applied to polymorphism research to accelerate solid-form discovery.

Polymorph evolution in glycine has been studied extensively by Harris and co-workers using ^13^C solid-state NMR (Hughes & Harris, 2008 ▸, 2009 ▸, 2010 ▸; Hughes *et al.*, 2015 ▸; Harris *et al.*, 2017 ▸). Harris’s work led us to select the same system for the present study. Glycine, which is the simplest amino acid, has six different polymorphs. Three polymorphs are known under ambient conditions. The α-form is monoclinic (*P*2_1_/*n*, *Z* = 4), the β-form is also monoclinic (*P*2_1_, *Z* = 2) and the γ-form is trigonal (*P*3_1_/*P*3_2_, *Z* = 3). The other forms (δ, ∊ and ζ) occur at high pressure. Glycine is in the zwitterionic form in all cases (^+^H_3_N–CH_2_–COO^−^), and all contain hydrogen-bonded head-to-tail chains of glycine molecules along [001]; the polymorphs differ in the way the chains pack together. The order of stability under ambient conditions is β < α < γ (Perlovich *et al.*, 2001 ▸; Boldyreva *et al.*, 2003 ▸). The crystallographic parameters for each phase are available in the supporting information (Table S1).

α-Glycine is obtained directly from aqueous solution. The γ-form has been obtained using a number of different methods including laser-assisted nucleation (Sun *et al.*, 2006 ▸; Liu *et al.*, 2017 ▸) and slow crystallization from a basic solution, but can also be obtained directly from aqueous solution in the case of the deuterated isotopologue (Hughes & Harris, 2009 ▸). β-Glycine is obtained by the addition of methanol/ethanol to a saturated glycine solution (Weissbuch *et al.*, 2005 ▸). Further work on β-glycine and techniques for obtaining it are provided in the supporting information.

## 2. Experimental   

A saturated solution of glycine (2.3857 g, Sigma–Aldrich ACS reagent ≥98.5%) in deionized water (9.3914 g) was filtered under gravity to remove any undissolved glycine. 3 µl aliquots of the solution were pipetted onto a TEM grid (Quantifoil R3.5/1) and allowed to stand at ambient conditions (298 K, 21% humidity). The water was removed by pressure-assisted blotting (Zhao *et al.*, 2019 ▸) at 3, 4 and 5 min and the sample immediately vitrified in liquid ethane to arrest further crystallization and protect the sample from beam and vacuum damage when under the microscope. A figure summarizing the procedure is available in the supporting information (Fig. S3). Rapid blotting was accomplished using a disk of filter paper secured with a rubber band over the top of a Büchner flask connected to a water aspirator. Glycine solution (3 µl) was also crystallized on a glass slide, ground using a pestle and mortar, dispersed onto a TEM grid (Quantifoil R2/2) and vitrified. Prior to freezing, the cryoTEM grids were plasma treated using an Easiglow discharge cleaning system for 45 s.

3DED data were collected on a Jeol JEM-2100 LaB_6_ transmission electron microscope operating at 200 kV in selected area electron diffraction (SAED) mode and a hybrid detector (Timepix, 512 × 512 pixels, Amsterdam Scientific Instruments). A Gatan tomography cryoholder was used operating at −175°C. During the data collection, diffraction patterns of the crystallites were collected while rotating the specimen continuously with a rotation range between 46 and 102° (Nederlof *et al.*, 2013 ▸; Nannenga *et al.*, 2014 ▸; Gemmi *et al.*, 2015 ▸; Wang *et al.*, 2017 ▸, 2018 ▸). The exposure time (0.3 s) and rotation speed (1.13° s^−1^) were chosen so that individual diffraction images were integrated over 0.34° of reciprocal space. The patterns were indexed with *REDp* (Wan *et al.*, 2013 ▸) and integrated with *XDS* (Kabsch, 2010 ▸). The structures were solved using *SHELXT* (Sheldrick, 2015*a* ▸) and refined using *SHELXL* (Sheldrick, 2015*b* ▸) through the *OLEX2* interface (Dolomanov *et al.*, 2009 ▸).

## 3. Results   

We have studied the sequence of polymorph formation during the *in situ* crystallization of glycine on a TEM grid from a saturated aqueous solution. The use of cryoTEM and 3DED has enabled the process to be studied at shorter timescales than has hitherto been possible. A drop of the solution was placed on a TEM grid and allowed to stand at ambient temperature for 3, 4 and 5 min.

**Figure 1:**
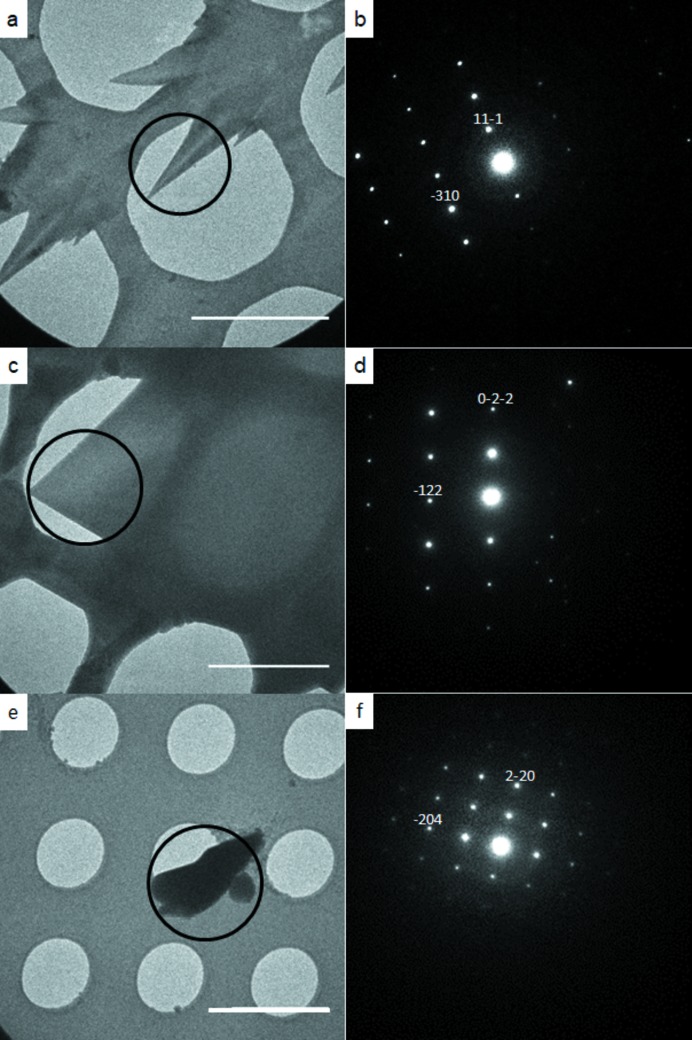
CryoTEM images (*a*), (*c*) and (*e*) with corresponding diffraction patterns (*b*), (*d*) and (*f*). (*a*) and (*b*) show β-glycine after 3 min of crystallization; (*c*) and (*d*) show α-glycine after 4 min; and (*e*) and (*f*) show γ-glycine after crystallization on a glass slide. The black circles on (*a*), (*c*) and (*e*) indicate the part of the crystal where the diffraction pattern was measured. Indexed reflections are shown on the diffraction patterns (*b*), (*d*) and (*f*). Scale bars: 3 µm. 2D slices from the 3D reciprocal lattices of selected datasets are provided in the supporting information.

**Figure 2:**
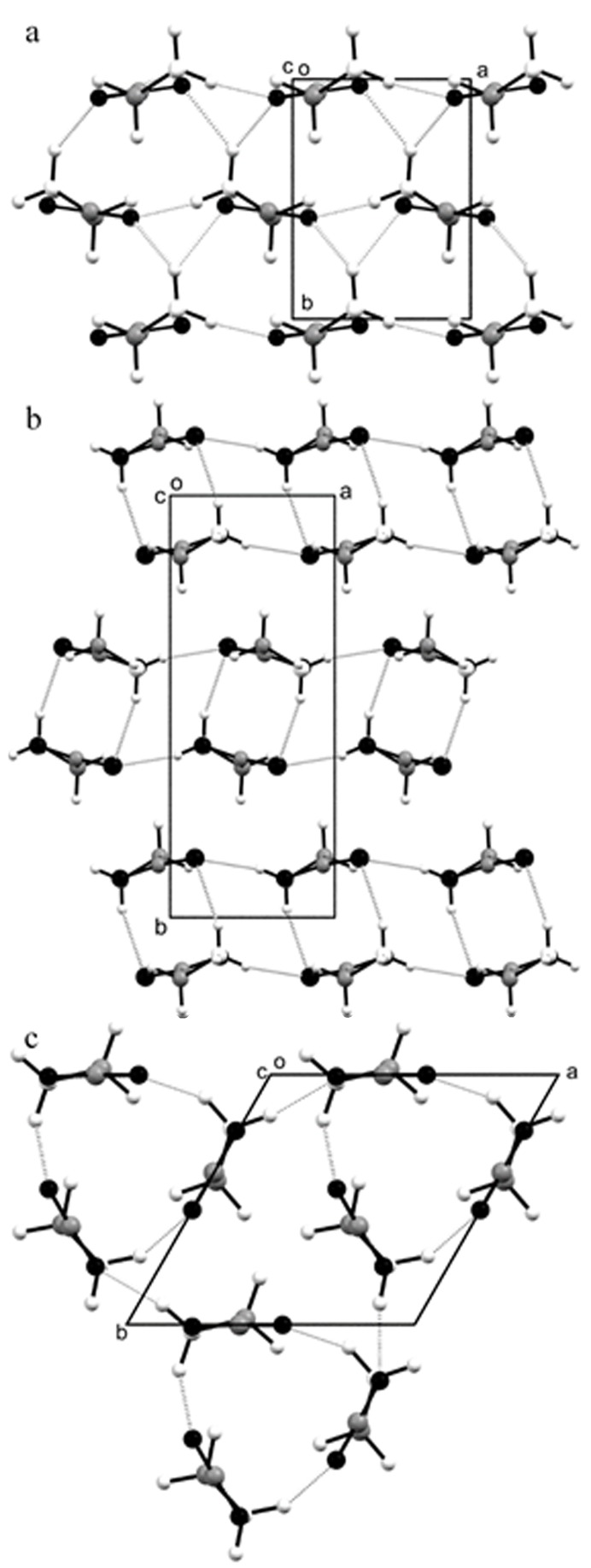
Crystal structures of (*a*) β-glycine, (*b*) α-glycine and (*c*) γ-glycine determined from 3DED data. All views are along the *c* axis. NH⋯O hydrogen bonds are shown as dotted lines. C – grey, O – black, N and H – white.

After 3 min, the grid was entirely populated by crystallites with a ‘shark’s tooth’ morphology, shown in Fig. 1 ▸(*a*). The crystals were of typical dimensions 2.5 µm × 0.5 µm in the plane of the images. 3DED data were collected on these crystallites using the continuous rotation method (Nederlof *et al.*, 2013 ▸; Nannenga *et al.*, 2014 ▸; Gemmi *et al.*, 2015 ▸; Wang *et al.*, 2017 ▸, 2018 ▸). The polymorph was identified as β-glycine from the unit-cell dimensions determined from the 3DED data [Fig. 1 ▸(*b*), with axial diffraction images available in Fig. S4 in the supporting information]. The diffraction images from seven crystallites were integrated and combined to give a single data set suitable for structure solution and refinement (Table S2 summarizes the crystallographic information for the datasets used for data merging). The crystal structure was solved by dual-space methods and refined by least-squares using the kinematic approximation, that is, in the same way that a conventional single-crystal X-ray diffraction data set would have been treated. The final *R* factor was 13%; the structure is shown in Fig. 2 ▸(*a*).

After 4 min, plate-like α-glycine crystals were observed in addition to the β-form. The α-glycine crystals were bigger (>3 µm) and had grown over the surface of the grid [Fig. 1 ▸(*c*)]. Both α- and β-glycine exhibited readily distinguishable morphologies, as shown in Figs. S1(*a*) and S1(*b*).

After 5 min, the α-glycine crystals were larger (5–10 µm) and thicker. Some β-glycine crystallites were also present [Figs. S2(*a*) and S2(*b*)]. Integrated 3DED data of α-glycine from six crystals from the 4 and 5 min samples were merged to form a data set suitable for structure determination [Fig. 1 ▸(*d*), axial diffraction images are given in Fig. S5]. The structure [Fig. 2 ▸(*b*)] was solved and refined as described above; the *R* factor was 22%.

In order to investigate a longer time scale, a 3 µl drop was allowed to evaporate to dryness over the course of 1 h on a glass slide, and then ground to ensure that the crystallites were small enough for electron diffraction patterns to be collected. Most of this sample was α-glycine, in the presence of some of the β-form (a listing of the unit-cell dimensions of the crystallites investigated is given in Table S6). One crystallite with a rather indistinct morphology, shown in Fig. 1 ▸(*e*), had unit-cell parameters, determined from 3DED data, of *a* = 7.44, *b* = 7.35, *c* = 5.75 Å, α = 89.21, β = 90.80, γ = 118.88°, characteristic of γ-glycine [Fig. 1 ▸(*f*); for axial diffraction images see Fig. S6]. The structure was solved and refined using 3DED data from only one crystal to give an *R* factor of 31% [Fig. 2 ▸(*c*)].

## 4. Discussion and conclusions   

We have shown for the first time that all three polymorphs of glycine can form sequentially from the same aqueous solution. The β-form appears first, in accordance with Ostwald’s Rule of Stages, but after only 1 min this begins to yield the α-form, which then becomes dominant. These changes occur over the course of only 2 min. When the same process was first studied by ^13^C solid-state NMR, spectra were recorded at a rate of every 16 min (Harris *et al.*, 2017 ▸). This was not quite quick enough to capture the initial formation of the β-form, and only the α-form was seen in H_2_O, though when the solvent was changed to D_2_O a slow transformation from α to γ was also observed (Hughes & Harris, 2008 ▸). Further optimization of the technique led to the transient β-glycine polymorph being observed in the first 5 min when crystallizing from methanol/water (Hughes & Harris, 2010 ▸, Hughes *et al.*, 2015 ▸, Harris *et al.*, 2017 ▸). However, neither the β nor the γ polymorphs were observed to form from pure isotopically natural water as they were here.

The combination of 3DED with the techniques used for specimen preparation in cryoTEM has clear advantages that strongly complement existing methods in polymorphism research. First, it is very fast in terms of sample preparation, imaging and diffraction data collection. The strong interaction of electrons with crystalline matter (Henderson, 1995 ▸), which enables crystal structures to be obtained from very small crystallites (1 µm or less) in micro- or even nano-gram quantities, means that polymorphs can be identified after only a few minutes of *in situ* growth on a TEM grid. The sample preparation method used in this study deviates from the conventional depositing–blotting–plunging technique. We were able to remove the majority of the solution by suction and immediately plunge-froze the grid, stopping further crystal growth. Removal of the aqueous phase is not exhaustive, and a film of mother liquor remains on the crystallites, but the absence of a substantial matrix of ice embedding the crystals reduces the inelastic scattering of the electron beam whilst also minimizing radiation damage.

Secondly, the method enables individual crystallites to be studied selectively. Polymorphs frequently display distinct morphologies, as the images in Fig. 1 ▸ show. New polymorphs can thus potentially be identified by inspection of the TEM images, with rapid 3DED data collection permitting diffraction patterns to be collected from single specific crystallites in <20 s. A crystal structure can be obtained from just one crystallite, so that crystal forms of low abundance can be identified, albeit with lower precision than when data from several crystallites are merged.

When treated in the same way as X-ray diffraction data, the resulting structures show clearly the intermolecular interactions and molecular conformations that distinguish one polymorph from another. However, they are characterized by *R* factors in the range 10–30% (Table S3), while bond distances and angles may also deviate from their ideal values (Tables S4 and S5). This is because the very strength of the interaction between electrons and matter that enables the study of small crystallites carries with it the disadvantage that beams scattered from one set of Bragg planes can be re-scattered by other planes. This primary extinction effect leads to a breakdown of the kinematical model of diffraction which has been so successful in the analysis of X-ray diffraction patterns. Merging data collected from several crystallites can provide better precision, but Palatinus and co-workers have recently described the application of the more appropriate dynamical scattering model during structure refinement, improving both accuracy and precision (Palatinus *et al.*, 2015 ▸; Colmont *et al.*, 2016 ▸; Brázda *et al.*, 2019 ▸; Gemmi *et al.*, 2019 ▸). The methods are computationally demanding, but this work is clearly a major step forward in electron crystallography.

The third advantage of *in situ* crystallization is that it is very gentle and non-invasive, involving no physical manipulation of the crystallites. Organic crystals are soft and fragile and can easily degrade when subjected to grinding or even simple transfer from one sample holder to another. Physical manipulation, which can also induce phase transitions, is thus avoided. The procedure ensures that no dehydration, and hence possible artefacts such as recrystallization caused by drying, take place. The non-invasive nature of *in situ* crystallization leads to high-quality images both in direct and reciprocal space.

Electron diffraction is one of the most rapidly developing and exciting areas of crystallography. The publication of a number of recent papers describing its application in chemical crystallography has led to a great deal of comment and anticipation in the chemical community. The present methods show that it can be applied to study dynamical chemical processes. Although we have focused on polymorphism, the same methods might also be applicable to reaction mixtures.

## 5. Related literature   

The following references are cited in the supporting information: Bernal (1931 ▸); Bouchard *et al.* (2007 ▸); Bull *et al.* (2017 ▸); Chongprasert *et al.* (2001 ▸); Dang *et al.* (2009 ▸); Dawson *et al.* (2005 ▸); Devi *et al.* (2014 ▸); Drebushchak *et al.* (2002 ▸); Ferrari *et al.* (2003 ▸); Fischer (1905 ▸); Hamilton *et al.* (2008 ▸); Han *et al.* (2013 ▸); Itaka (1960 ▸); Lee *et al.* (2008 ▸); Nishijo & Kinigusa (1973 ▸); Pyne & Suryanarayanan (2001 ▸); Seyedhosseini *et al.* (2014 ▸); Torbeev *et al.* (2005 ▸); Xu *et al.* (2017 ▸).

## Supplementary Material

Crystal structure: contains datablock(s) alpha, beta, gamma. DOI: 10.1107/S2052252519016105/yc5021sup1.cif

Supporting tables and figures. DOI: 10.1107/S2052252519016105/yc5021sup2.pdf
