# Supplementary material for: Polymorph evolution during crystal growth studied by 3D electron diffraction
Source: IUCrJ. 2020 Jan 1;7(Pt 1):5–9. doi: 10.1107/S2052252519016105 (PMC6949601; doi:10.1107/S2052252519016105)
Supplement: Supplementary file 2 [file m-07-00005-sup2.pdf]

IUCrJ

Volume 76 (2020)

Supporting information for article:

Polymorph evolution during crystal growth studied by 3D electron diffraction

Edward T. Broadhurst, Hongyi Xu, Max T. B. Clabbers, Molly Lightowler, Fabio Nudelman, Xiaodong Zou and Simon Parsons

Supporting information

Polymorph evolution during crystal growth studied by 3D electron diffraction

1. Crystallisation of β glycine

2. Supplementary Tables

Table S1: Crystallographic Information on the six different polymorphs of glycine as well as another solid phase of glycine, glycine dihydrate (GDH).

Table S2: Summary of the crystallographic information used for the structure solution of α and β glycine.

Table S3: Experimental crystallographic data and refinement data. Experiments were carried out with electron radiation, λ = 0.02508 Å.

Table S4: Bond Lengths (Å) and Angles (deg) for α, β, and γ-glycine from CSD refcodes GLYCIN29, GLYCIN31 and GLYCIN33 respectively.

Table S5: Bond Lengths (Å) and Angles (deg) for α, β, and γ-glycine from experimental ED data.

Table S6: Unit cell data from the glycine sample after prolonged standing.

3. Supplementary Figures

Figure S1: Low magnification image of a) α and b) β glycine after 4 minutes. The crystals of β glycine exhibit a shark tooth like morphology in the holes of the TEM grid. α glycine grows across the grid and is larger than the β glycine crystals. The small dark particles are ice crystals formed during the cryo-transfer of the TEM grid. Scale bars = 3μm.

Figure S2: Low magnification images after 5 minutes showing a) darker and more block-like α glycine. b) Higher magnification image area in a marked by the red box showing crystals of β glycine, next to α glycine RHS showing distinctly different morphology.

Figure S3: Diagram illustrating the method of sample preparation for the glycine samples for TEM.

Figure S4 - S6: 2D slices from the 3D reciprocal lattices viewed along different axes for beta, alpha and gamma.

4. References

IMPORTANT: this document contains embedded data - to preserve data integrity, please ensure where possible that the IUCr Word tools (available from http://journals.iucr.org/services/docxtemplate/) are installed when editing this document.

1. Crystallisation of β glycine

Fischer (Fischer, 1905) was the first to observe β glycine by addition of ethanol to a saturated aqueous solution. The crystal structure was first reported by Itaka (Itaka, 1960). Since then, a large volume of literature has been published investigating the different conditions needed to promote formation of this metastable form. The first, widely used method, involved addition of ethanol/methanol to a concentrated aqueous solution of glycine at neutral pH (Perlovich *et al.*, 2001, Fischer, 1905, Itaka, 1960, Bernal, 1931). This was used in work to monitor β glycine as a transient polymorph during crystallisation in situ via ^{13}C NMR (Hughes & Harris, 2010).

Other work described the use of a 1:2 mixture of water and glacial acetic acid which was left to stand for 3 days. In one of the crystallisations, nearly 1 g of pure β glycine was produced (Boldyreva *et al.*, 2003). This method was modified for use calorimetric investigations (Drebushchak *et al.*, 2002), where a saturated solution of glycine in a mixture of water and glacial acetic acid (ratio 5:1) was prepared then filtered. Acetone in equal volume was added to the filtered solution in a ratio of 1:1 and the turbid solution filtered. An equal volume of ethanol could also be used (Dang *et al.*, 2009, Bouchard *et al.*, 2007, Ferrari *et al.*, 2003). Formation of β glycine is the result of an increased concentration of solvated glycine monomers relative to the hydrogen bonded dimers which are a feature of the α polymorph (Weissbuch *et al.*, 2005).

Thermal decomposition of aminomalonic acid with glycine has been claimed to lead to formation of β glycine due to the slow rate of decomposition during which the hydrogen bonding remains intact, inducing the formation of the unstable polymorph (Nishijo & Kinigusa, 1973). During slow cooling of glycine-water mixtures, only a β glycine/ice eutectic mixture is formed, melting at −3.60°C (Chongprasert *et al.*, 2001).

A study on the phase transitions of glycine in frozen aqueous solutions and during freeze drying found that β glycine crystallises from a phase later identified as glycine dihydrate at −70 °C (Pyne & Suryanarayanan, 2001, Xu *et al.*, 2017). Devi et al. describe a way in which the sudden cooling of aqueous glycine solutions from 353 K to 273 K (not cold enough to freeze the solution) yielded the β polymorph (Devi *et al.*, 2014).

An *in situ* study using Raman spectroscopy to determine the effect of the pH on the observed outcome of glycine crystallisation showed that moderate acidic/basic conditions (pH = 4.5 – 8.5) accelerated the nucleation of α glycine, whilst in extreme acidic/basic (pH<4.5 or >8.5) conditions, only γ glycine was observed. A peak of 1324 cm^{-1} was observed for the α-form crystallised from pure, additive-free aqueous solution; whilst in an acidic solution, a peak of 1337 cm^{-1}, indicating the γ-form was measured. A peak at 1321 cm^{-1}, corresponding to the β-form, was never seen and it was suggested that the β-form had already transformed in the time taken to record the spectra (Han *et al.*, 2013).

The presence of enantiopure α-amino acids with bulky side groups such as racemic tryptophan (Trp), N-CH_3-Trp, and R-naphthylalanine cause β-glycine to crystallise from aqueous solution (Torbeev *et al.*, 2005). Crystallisation of glycine by evaporation of aqueous solutions in nano-sized chambers favours β glycine (Hamilton *et al.*, 2008) and the sample remains stable for up to a year within the nano-sized chambers.

The α, β and γ-forms were observed during crystallisation from acidic, neutral and basic solutions of various concentrations on a patterned, gold-substrate. The substrate contained roughly 2000 islands with dimensions of 500 μm. In general, β-glycine was observed more at lower concentrations across the range of pHs (3.4 - 10.1) (Lee *et al.*, 2008). In related work, Seyedhosseini *et al.* grew stable microcrystals of β-glycine on a (111)Pt /SiO_2 /Si substrate (Seyedhosseini *et al.*, 2014). The solutions ranged from 0.0133 – 1.77 M and β-glycine was observed across all crystallisations. The crystals were characterised by XRD and Raman spectroscopy and some β-glycine was stable on the Pt substrate for up to one month.

2. Supplementary Tables.

Table S1 Crystallographic Information on the six different polymorphs of glycine as well as another solid phase of glycine, glycine dihydrate (GDH).

Polymorph	α	β	γ	δ	ε	ζ	GDH
Space group	$P2_1/n$	$P2_1$	$P3_1$	$P2_1/a$	Pn	$I1$	$P2_1/c$
a/Å	5.1047(3)	5.0932(16)	7.0383(7)	11.156(4)	4.8887(10)	5.1029 (16)	8.9585(9)
b/ Å	11.9720(14)	6.272(3)	7.0383(7)	5.8644(11)	5.7541(11)	6.3450 (12)	8.2166(8)
c/ Å	5.4631(3)	5.3852(18)	5.4813(8)	5.3417(17)	5.4419(11)	5.4331 (18)	7.6142(6)
alpha/ °	90	90	90	90	90	85.91 (3)	90
beta/ °	111.740(5)	113.19(3)	90	125.83(4)	116.682(10)	114.26 (3)	104.262(7)
gamma/ °	90	90	120	90	90	103.55 (3)	90
V/ Å^3	310.10(4)	158.13(9)	235.15	283.32(16)	136.78(5)	155.85 (9)	543.20(8)
Temperature/ K	298	298	298	298	298	298	208
Pressure/ GPa	0	0	0	1.9	4.3	0	0
REFCODE	GLYCIN29	GLYCIN31	GLYCIN33	GLYCIN67	GlYCIN68	DOLBIR43	BAHMAB
Reference	Boldyreva et al. 2003(Boldyreva *et al.*, 2003)	Boldyreva et al. 2003(Boldyreva *et al.*, 2003)	Boldyreva et al. 2003(Boldyreva *et al.*, 2003)	Parsons et al. 2005(Dawson *et al.*, 2005)	Parsons et al. 2005(Dawson *et al.*, 2005)	Bull et al. 2017(Bull *et al.*, 2017)	Xu et al. 2017(Xu *et al.*, 2017)

Table S2 Summary of the crystallographic information used for the structure solution of α and β glycine.

Dataset	*a*/Å	*b*/Å	*c*/Å	β/º	*V*/Å^3	Polymorph
3 minutes						
13	5.563	6.784	5.978	111.97	209.2	β
20	5.310	6.410	5.730	113.10	179.4	β
22	5.246	6.495	5.690	114.02	177.1	β
24	5.374	6.511	5.683	112.50	183.7	β
33	5.232	6.542	5.649	113.75	177.0	β
35	5.358	6.376	5.636	113.14	177.1	β
39	5.503	6.402	5.710	114.19	183.5	β
4 minutes						
4	5.193	12.363	5.617	110.32	338.2	α
5	5.199	12.395	5.625	110.53	339.5	α
7	5.206	12.443	5.623	110.62	340.9	α
5 minutes						
4	4.959	11.658	5.425	112.63	289.5	α
5	5.259	12.789	5.765	111.93	359.7	α
7	5.263	12.543	5.772	111.83	353.7	α

Table S3 Experimental crystallographic data and refinement data. Experiments were carried out with electron radiation, $\lambda = 0.02508$ Å.

Polymorph	β	α	γ
Crystal system	Monoclinic	Monoclinic	Trigonal
Space group	$P2_1$	$P2_1/n$	$P3_1$
a, *b*, *c* (Å)	5.3110 (11), 6.4540 (13), 5.6940 (11)	5.223 (1), 12.435 (3), 5.5630 (11)	7.395 (1), 7.395 (1), 5.7500 (12)
α, β, γ (°)	90, 112.86 (3), 90	90, 111.14 (3), 90	90, 90, 120
V (Å^3)	179.84 (7)	336.99 (13)	272.32 (9)
Z	2	4	3
Crystal size (µm)	2.5	>3	2 - 2.5
Data collection			
No. of measured, independent and observed reflections [Fo > 4sig(Fo)]	2516, 859, 745	5404, 850, 561	769, 625, 160
R_{int}	0.209	0.318	0.248
Resolution (Å)	0.751	0.703	0.700
Completeness (%)	97	85	61
Refinement			
$R1$ (calculated over all amplitudes for all reflections), $wR2$, S	0.128, 0.296, 1.17	0.219, 0.518, 1.04	0.306, 0.630, 1.01
No. of reflections	859	850	625
No. of parameters	54	51	18
No. of restraints	1	24	9
H-atom treatment	All H-atoms found in residual density and constrained	All H-atoms found in residual density and constrained	All H-atoms found in residual density and constrained
Absolute structure	All f'' are zero, so absolute structure could not be determined	N/A	All f'' are zero, so absolute structure could not be determined

Table S4 Bond Lengths (Å) and Angles (deg) for α, β, and γ-glycine from CSD structures refcodes GLYCIN29, GLYCIN31 and GLYCIN33 respectively.

Bond Lengths	α	β	γ
O1-C2	1.2550(11)	1.2527(12)	1.250(4)
O2-C2	1.2518(10)	1.2528(12)	1.258(4)
N1-C1	1.4778(11)	1.4752(14)	1.470(4)
C1-C2	1.5269(10)	1.5283(13)	1.526(4)

Bond Angles	α	β	γ
O1-C2-O2	125.63(7)	125.77(8)	125.7(3)
O1-C2-C1	116.93(7)	117.03(7)	116.8(3)
O2-C2-C1	117.44(7)	117.15(8)	117.5(3)
N1-C1-C2	111.70(6)	111.80(7)	111.7(2)

Table S5 Bond Lengths (Å) and Angles (deg) for α, β, and γ-glycine from experimental ED data.

Bond Lengths	α		β		γ
N1-C1	1.509(6)	N1-C1	1.535(6)	N1-C1	1.45(2)
C1-C2	1.566(6)	C1-C2	1.600(9)	C1-C2	1.491(19)
C2-O2	1.275(6)	C2-O2	1.320(6)	C2-O2	1.30(2)
C2-O1	1.304(6)	C2-O1	1.323(8)	C2-O1	1.28(2)
Bond Angles					
N1-C1-C2	111.1(4)	N1-C1-C2	112.4(5)	N1-C1-C2	116.0(17)
O2-C2-O1	126.2(4)	O2-C2-O1	124.6(6)	O2-C2-O1	122(2)
O2-C2-C1	117.0(4)	O2-C2-C1	117.7(6)	O2-C2-C1	120.4(18)
O1-C2-C1	116.8(4)	O1-C2-C1	117.7(4)	O1-C2-C1	117.2(16)

Table S6 Complete unit cell data of datasets from the glycine sample after prolonged standing. Unit cells were determined with cRED and are unrefined. Entries with * in the last column are probably twinned.

a/Å	*b*/Å	*c*/Å	α/°	β/°	γ/°	*V*/Å^3	Polymorph
50.75	39.85	17.72	59.03	119.53	16.44	10283.70	*
7.64	4.68	4.63	119.11	90.16	90.20	144.80	ice
35.96	55.73	44.06	141.59	92.18	114.51	38253.50	*
12.43	5.56	5.23	111.48	90.93	89.97	336.20	α
8.26	4.26	4.66	120.52	90.83	91.02	153.30	ice
12.38	5.21	5.57	111.94	90.40	89.47	333.80	α
6.40	5.44	5.28	112.01	93.04	88.95	170.30	β
12.13	5.67	5.17	111.45	89.62	90.82	330.80	α
9.25	4.68	8.76	122.01	56.66	107.07	146.30	*
7.74	4.66	4.61	118.94	89.34	92.01	145.00	ice
40.79	26.62	6.17	102.75	49.44	162.21	1988.80	*
10.57	5.51	5.43	119.26	87.32	95.39	274.90	α
10.44	5.41	5.51	119.20	93.13	91.79	270.90	α
12.11	5.61	5.28	112.86	89.20	90.51	329.80	α
27.40	33.54	30.02	155.49	114.50	68.35	10408.50	*
6.31	5.26	5.49	113.55	91.39	88.41	166.90	β
11.86	5.56	5.32	111.95	90.49	90.02	325.20	α
21.85	9.09	16.66	95.60	127.97	105.18	2367.60	*
29.97	18.26	9.56	138.20	45.26	153.60	1539.60	*
7.47	4.68	4.62	119.18	89.25	90.64	141.10	ice
8.92	4.69	4.63	119.18	90.24	89.99	168.90	ice
7.78	4.68	4.68	119.27	92.18	88.20	148.40	ice
7.59	4.68	4.65	119.15	88.65	91.18	144.20	ice
7.59	4.69	4.70	120.00	90.54	91.02	144.80	ice
14.37	5.83	5.17	102.67	88.94	109.28	398.60	*
8.94	5.55	8.81	54.60	131.83	126.12	250.30	*

7.26	7.23	5.67	90.80	90.55	119.06	260.30	γ
7.40	5.75	7.35	90.67	118.68	89.54	274.30	Repeat of above
13.26	8.43	83.31	90.00	90.00	131.84	6940.50	*
7.75	4.67	4.64	118.73	89.80	90.16	147.30	ice
7.71	4.60	4.73	118.06	91.11	89.44	148.00	ice
17.54	19.15	12.23	136.04	51.40	146.31	1577.40	*
7.64	4.69	4.73	119.67	89.76	91.17	147.50	ice

3. Supplementary Figures

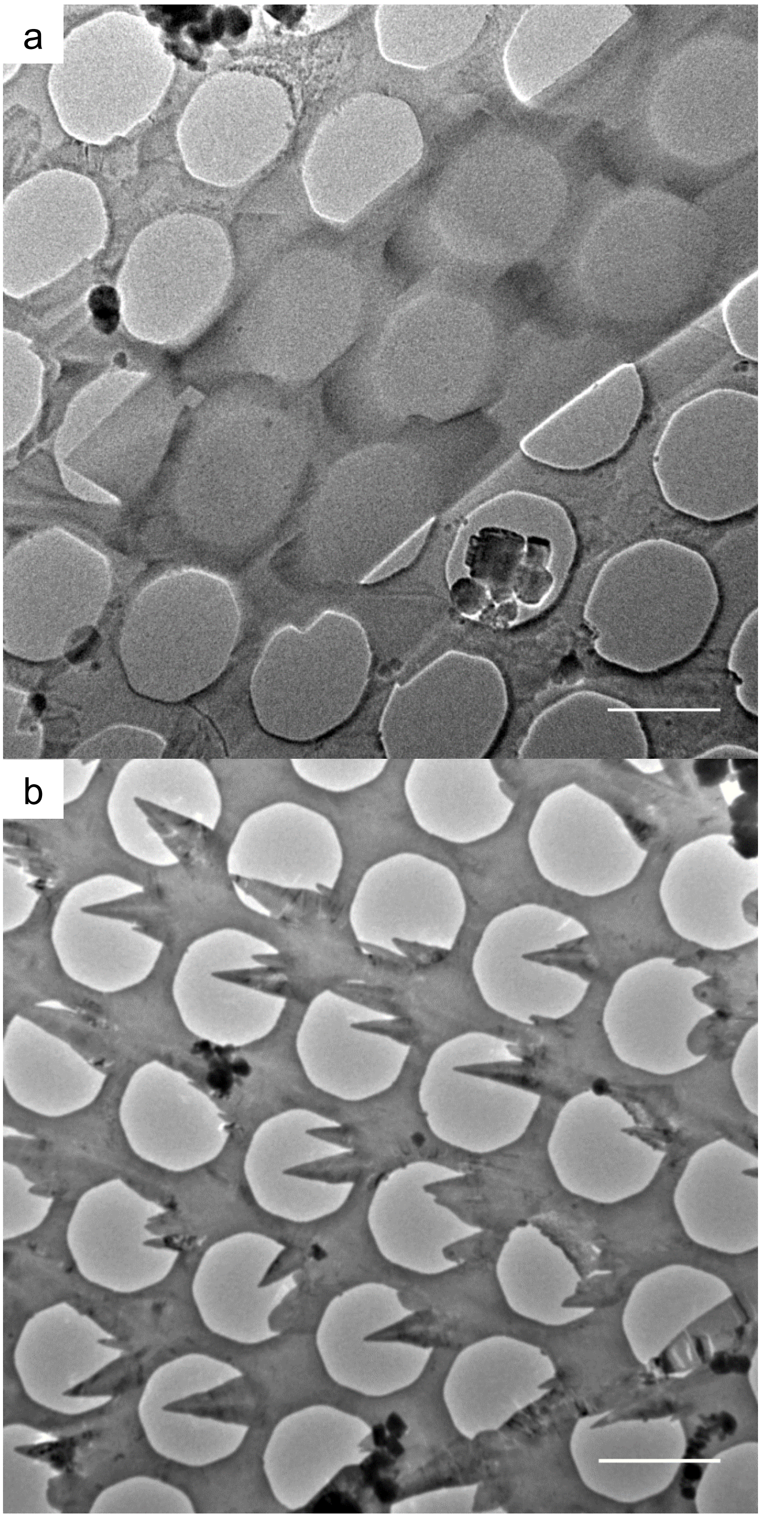

Figure S1 Low magnification image of a) α and b) β glycine after 4 minutes. The crystals of β glycine exhibit a shark tooth like morphology in the holes of the TEM grid. α glycine grows across the grid and is larger than the β glycine crystals. The small dark particles are ice crystals formed during the cryo-transfer of the TEM grid. Scale bars = 3μm.

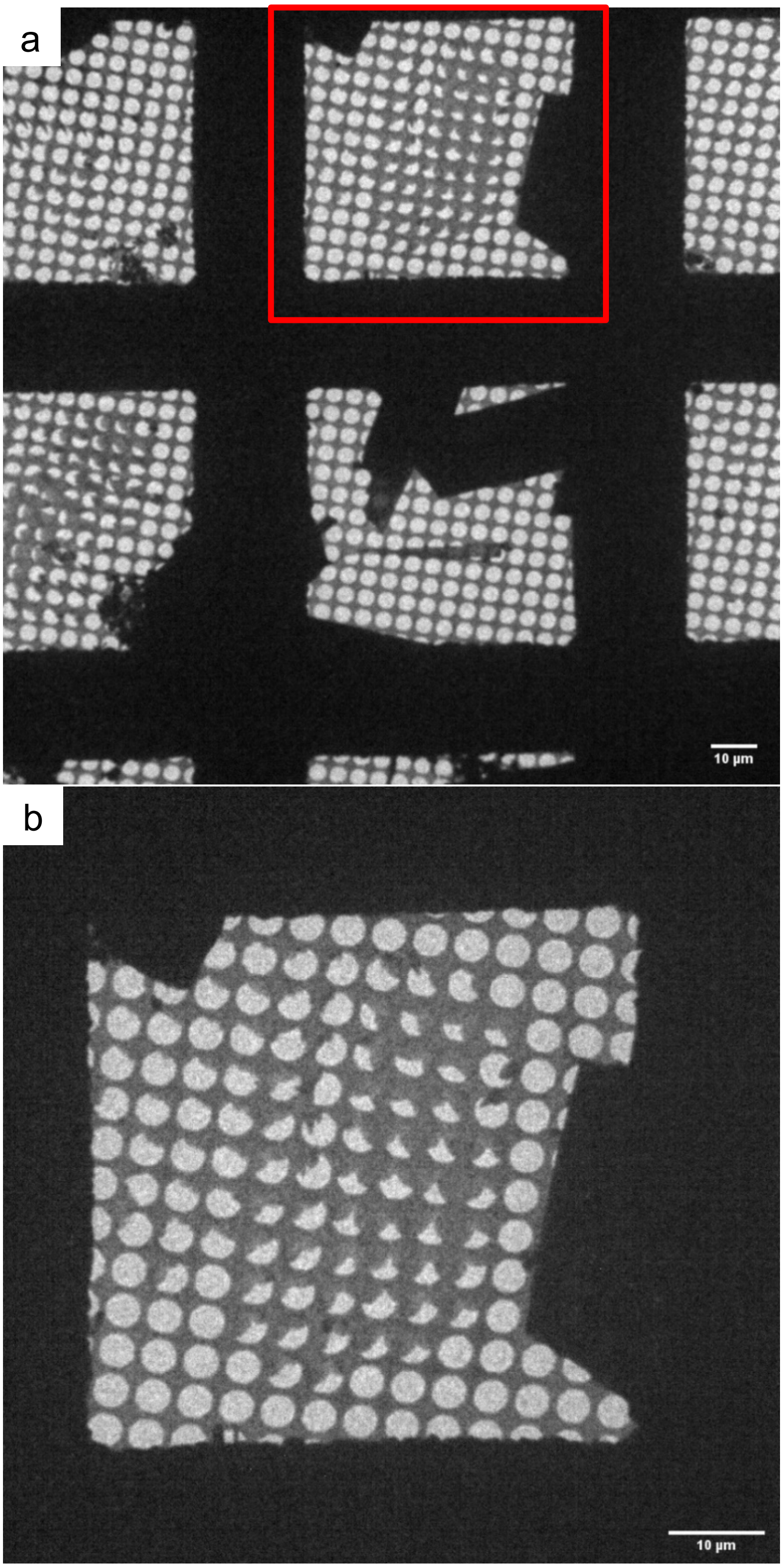

Figure S2 Low magnification images after 5 minutes showing a) darker and more block-like α glycine. b) Higher magnification image area in a marked by the red box showing crystals of β glycine, next to α glycine RHS showing distinctly different morphology.

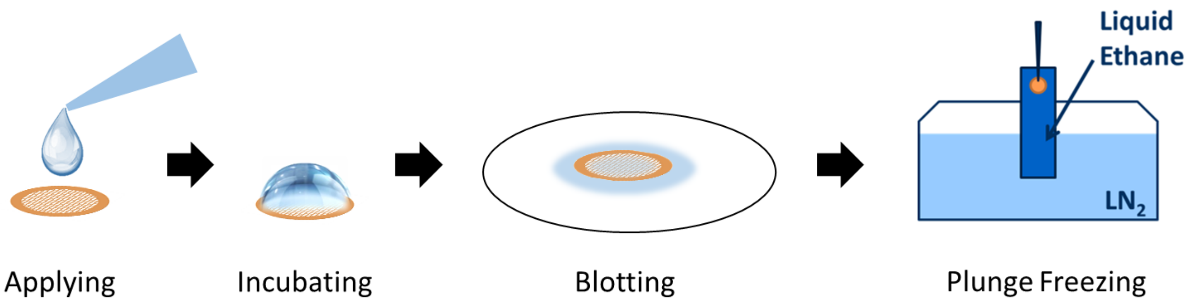

Figure S3 Diagram illustrating the method of sample preparation for the glycine samples for TEM.

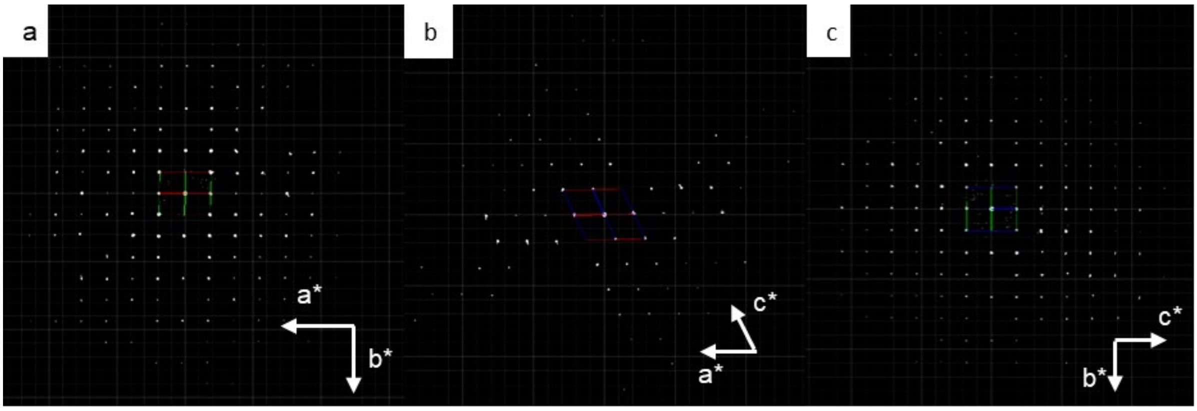

Figure S4 2D slices from the 3D reciprocal lattice of beta glycine from the 4 minute sample. a) shows dataset 20 with the hk0 plane viewed along the c* axis. b) shows dataset 22 with the h0l plane viewed along the b* axis. c) shows dataset 39 with the 0kl plane viewed along the a* axis. The reflection condition at 0k0 is k = 2n and therefore the space group is $P2_1$.

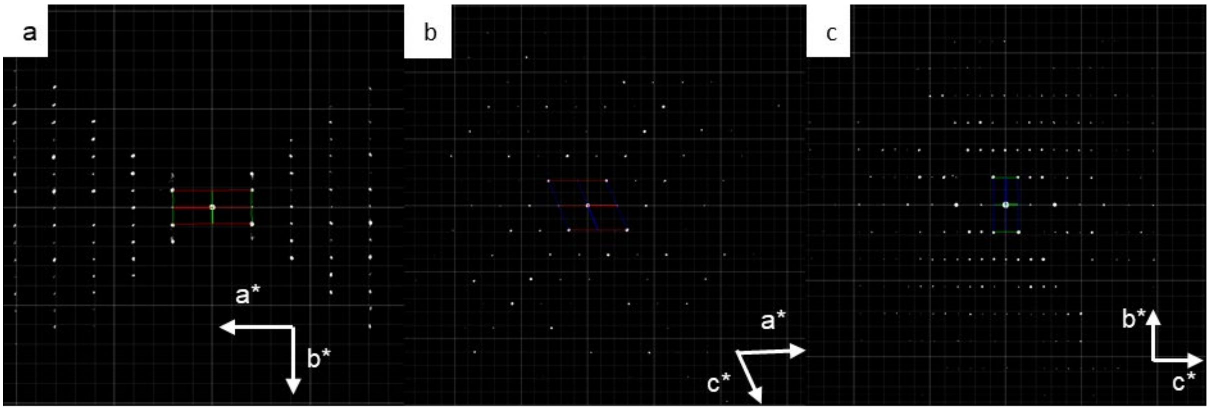

Figure S5 2D slices from the 3D reciprocal lattice of alpha glycine from the 5 and 6 minute sample. a) shows dataset 4 at 5 minutes with the hk0 plane viewed along the c* axis. b) shows dataset 4 at 5 minutes with the h0l plane viewed along the b* axis. c) shows dataset 9 at 6 minutes with the 0kl plane viewed along the a* axis. The reflection conditions at 0k0 and h0l are k = 2n and h+l = 2n therefore the space group is $P2_1/n$.

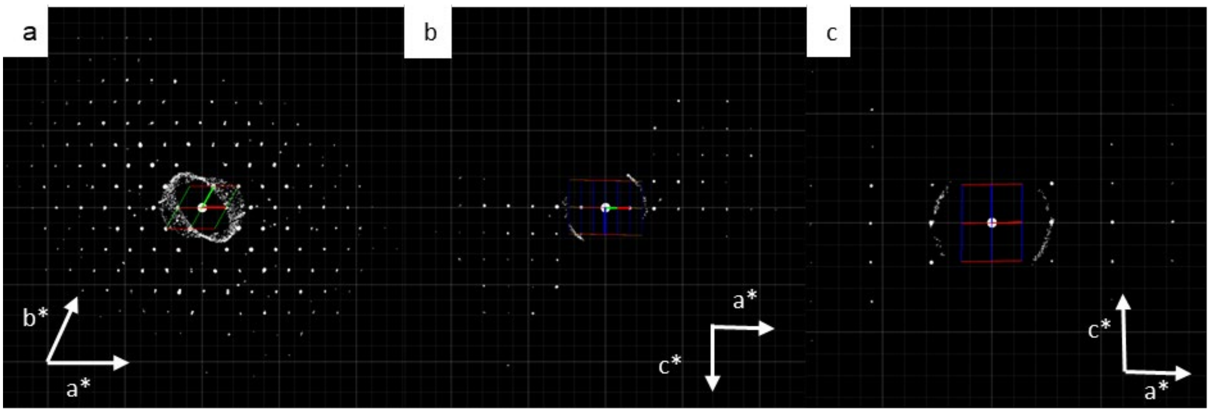

Figure S6 2D slices from the 3D reciprocal lattice of gamma glycine from the ground sample. a) shows the 3D reciprocal lattice viewed along the c* axis. b) shows the h0l plane viewed along the b* axis. c) shows the 2h-hl plane viewed along the a* axis. This is a primitive cell and contains no c glide. Based on the literature this is space group $P3_1$.

4. References

Bernal, J. D. (1931). *Z. Krist.* **78**, 363-369.

Boldyreva, E. V., Drebushchak, T. & Shutova, E. (2003). *Z. Krist.* **218**, 366.

Bouchard, A., Hofland, G. W. & Witkamp, G.-J. (2007). *J. Chem. Eng. Data* **52**, 1626-1629.

Bull, C. L., Flowitt-Hill, G., de Gironcoli, S., Küçükbenli, E., Parsons, S., Huy Pham, C., Playford, H. Y. & Tucker, M. G. (2017). *IUCrJ* **4**, 569-574.

Chongprasert, S., Knopp, S. A. & Nail, S. L. (2001). *J. Pharm. Sci.* **90**, 1720-1728.

Dang, L., Yang, H., Black, S. & Wei, H. (2009). *Organic Process Research & Development* **13**, 1301-1306.

Dawson, A., Allan, D. R., Belmonte, S. A., Clark, S. J., David, W. I. F., Mcgregor, P. A., Parsons, S., Pulham, C. R. & Sawyer, L. (2005). *Cryst. Growth Des.* **5**, 1415-1427.

Devi, K. R., Gnanakamatchi, V. & Srinivasan, K. (2014). *J. Cryst. Growth* **400**, 34-42.

Drebushchak, V. A., Boldyreva, E. V., Drebushchak, T. N. & Shutova, E. S. (2002). *J. Cryst. Growth* **241**, 266-268.

Ferrari, E. S., Davey, R. J., Cross, W. I., Gillon, A. L. & Towler, C. S. (2003). *Cryst. Growth Des.* **3**, 53-60.

Fischer, E. (1905). *Ber. Dtsch. Chem. Ges.* **38**, 2917.

Hamilton, B. D., Hillmyer, M. A. & Ward, M. D. (2008). *Cryst. Growth Des.* **8**, 3368-3375.

Han, G., Thirunahari, S., Shan Chow, P. & H Tan, R. B. (2013). *CrystEngComm* **15**, 1218.

Hughes, C. E. & Harris, K. D. M. (2010). *Chem. Comm.* **46**, 4982-4984.

Itaka, Y. (1960). *Acta Cryst* **13**, 35.

Lee, I. S., Kim, K. T., Lee, A. Y. & Myerson, A. S. (2008). *Cryst. Growth Des.* **8**, 108-113.

Nishijo, J. & Kinigusa, T. (1973). *Bull. Chem. Soc. Japan* **46**, 1003-1004.

Perlovich, G. L., Hansen, L. K. & Bauer-Brandl, A. (2001). *Journal of Thermal Analysis and Calorimetry* **66**, 699-715.

Pyne, A. & Suryanarayanan, R. (2001). *Pharm. Res.* **18**, 1448-1454.

Seyedhosseini, E., Ivanov, M., Bystrov, V., Bdikin, I., Zelenovskiy, P., Shur, V. Y., Kudryavtsev, A., Mishina, E. D., Sigov, A. S. & Kholkin, A. L. (2014). *Cryst. Growth Des.* **14**, 2831-2837.

Torbeev, V. Y., Shavit, E., Weissbuch, I., Leiserowitz, L. & Lahav, M. (2005). *Cryst. Growth Des.* **5**, 2190-2196.

Weissbuch, I., Torbeev, V. Y., Leiserowitz, L. & Lahav, M. (2005). *Angew. Chem. Int. Ed.* **44**, 3226-3229.

Xu, W., Zhu, Q. & Hu, C. T. (2017). *Angew. Chem. Int. Ed.* **129**, 2062-2066.
